# Signatures of immune cell infiltration for predicting immune escape and immunotherapy in cervical cancer

**DOI:** 10.18632/aging.204583

**Published:** 2023-03-13

**Authors:** Fuxing Chen, Lingzhi Shen, Ying Wang, Yaping Chen, Xuejiao Pan, Hui Liang, Hu Yu

**Affiliations:** 1 Zhejiang Provincial Center for Disease Control and Prevention, Institute of Immunization and Prevention, Hangzhou, China

**Keywords:** immune cell infiltration, immune escape, immunotherapy, cervical cancer

## Abstract

The cervical cancer tumor microenvironment is a diverse and complex ecosystem. Tumor-immune cell infiltration (ICI) may influence immune escape and immunotherapeutic responses. However, the relationship between immune cell infiltrations, immune escape, and immunotherapy in cervical cancer has not been fully clarified. Here, Principal component analysis (PCA) and Tumor immune dysfunction and exclusion (TIDE) were applied to calculate individual ICI scores and probabilities of immune escape, respectively. Through the IMvigor210 and the Cancer Immunome Atlas (TCIA) datasets, we validated the different responses to immunotherapy in two subgroups of patients. Furthermore, therapeutic benefits of different patients were predicted by the pRRophetic package. We found that patients with high ICI scores were prone to immune escape due to the activated JAK-STAT signaling pathway, along with lower CD8+ T cells. High ICI scores patients could benefit more from anti-PD-L1 immunotherapy, and individuals with low scores may be better candidates for the anti-CTLA-4 treatment. Combinations of immunotherapies with targeted inhibitors may improve clinical efficacy and reduce the risk of tumor recurrence. The ICI model not only helps us enhance the cognition of immune escape, but also guides the application of immunotherapy in cervical cancer patients.

## INTRODUCTION

Cervical cancer (CC) is a serious global health issue. Although the anti-HPV vaccine can effectively reduce CC incidence and mortality, vaccination does not treat HPV infection [1]. Chemotherapy, radiation, and surgery are currently used to treat early-stage CC [2]. However, patients with advanced and recurrent disease remains poor prognosis [2, 3].

Unlike traditional therapy, immunotherapy acts through mechanisms against cancer cells by modifying and recruiting the host's immune system. It aims to supplement and support existing immune cells in the tumor microenvironment (TME) [4, 5]. Tumor cells can induce immune escape by inhibiting immune cell function through PD-1/PD-L1 signaling, which influences cancer progression and therapeutic efficacy for immunotherapy [6, 7]. Immunotherapies targeting immune suppressive cells, such as Tregs and tumor-associated macrophages (TAMs), could reverse immunosuppressive environments and prevent further tumor growth [8]. Currently, immune checkpoint molecules of cytotoxic T-lymphocyte-associated antigen 4 (CTLA-4) and programmed cell death receptor (PD-1), as the most promising tumor immunotherapies, have received more attention in clinical applications [9, 10]. PD-1/PD-L1 axis could block immune cells biological activities, including CD8+ T cells [11], and promote cancer immune escape [12]. Blocking immune checkpoint function may enhance CD8+ T cell proliferation and promote the clearance of cancer cells [12]. Thus, immunotherapies against immune-suppressive factors can reverse immunosuppressive environments, inhibit cancer growth, and enhance the recognition of cancer cells by immune cells [13, 14]. Nonetheless, not all cancer patients could benefit equally from the interventions of immunotherapy, identification of potential immune escape mechanisms in cervical cancer patients will facilitate customized therapeutic regimens for different CC patients.

Here, we evaluated immune cell infiltration (ICI) and immune microenvironment in CC using ESTIMATE and CIBERSORT and identified three immune subtypes and gene subtypes. In addition, ICI-based scoring subgroups were established to predict the probability of immune escape and response to immunotherapy.

## MATERIALS AND METHODS

### CC datasets and samples

The TCGA dataset containing CC gene expression and clinical features, and a GSE44001dataset from GEO were utilized in this study. The pre-processed TCGA dataset included 3 normal samples and 306 tumor samples. The GSE44001 dataset was based on 300 cervical cancer samples. The FPKM expression values were then converted into the transcripts per kilobase million (TPMs). The “ComBat” algorithm was applied to the merged database to reduce the probability of batch effects caused by non-biological technical deviations between datasets.

### Consensus clustering for tumor-infiltrating immune cells

The CIBERSORT tool was used to analyze infiltration levels of 22 types of immune cells (LM22 signature) in each sample using 1,000 permutations. The contents of immune and stromal cells in CC were acquired by the ESTIMATE algorithm. Based on immune cell infiltration levels of each sample, each case was assigned to the corresponding cluster based on hierarchical agglomerative clustering. “ConsensuClusterPlus” in R was used to execute the “Pam” method based on Euclidean and Ward’s linkage with 1,000 repeats to guarantee classification stability.

### Identification of ICI phenotype-associated differentially expressed genes (DEGs)

All genes that were not counted in any of the samples were dropped. The infiltration levels of immune cells were used to assign the study subjects to 3 immune cell infiltration (ICI) clusters. To identify ICI phenotype-associated genes, the cutoff thresholds for fold change and false discovery rate (FDR) were set at |log2-fold change| >1 and adjusted *p*-value < 0.05, respectively.

### Reduction of dimension and ICI score generation

Unsupervised clustering was performed to cluster the samples based on DEGs. Also, DEGs with positive correlation with gene clusters were defined as gene type A and those negative as gene type B. To minimize redundant or noise genes, the Boruta algorithm was used for finding the feature genes, and the gene type A and B were dimensionally reduced through principal component analysis (PCA) analysis [15]. The main component 1 extracted from PCA was taken as a signature score [16]. Finally, an analogous was applied to evaluate the ICI score of each sample:


$$ICI\;score=\sum PC{\text{1}}_{A}-\;\sum PC{\text{1}}_{B}$$

### Collection of tumor mutation burden data

The mutation data of CC were acquired from the TCGA database. To evaluate tumor mutation burden in CC, we counted the non-synonymous mutations. Based on ICI scores, mutation data samples were classified into low or high ICI score groups. Mutation analysis and visualization were conducted using the maftools package.

### Evaluation of immunotherapy response

TIDE analysis was performed to calculate TIDE scores, an indicator of the probability of immune escape (http://tide.dfci.harvard.edu/). Moreover, an independent dataset which contained immunotherapy was obtained from IMvigor210 and clinical data were used to assess the therapeutic effectiveness of ICI (http://research-pub.gene.com/IMvigor210CoreBiologies/). In addition, the Cancer Immunome Atlas (TCIA; https://tcia.at/) was used to detect immune scores (IPS) of tumor samples to predict responses to cytotoxic T-lymphocyte antigen-4 (CTLA-4) and programmed cell death protein 1 (PD-1) blockers [17]. In this study, we also predicted the therapeutic sensitivity between high and low ICI score groups. The concentration (IC50) that leads to a 50% reduction growth of targeted inhibitors (TIs) was assessed with R package “pRRophetic” [18].

### Statistical analysis

All analyses were conducted using the R software. Kaplan–Meier analysis was performed to determine survival outcomes. Comparisons of survival differences among groups were carried out using the log-rank method. Significant differences were established at *P* < 0.05.

### Data availability

The supporting datasets were available from the public and described in the manuscript.

## RESULTS

### Immune cell infiltrations

**Figure 1:**
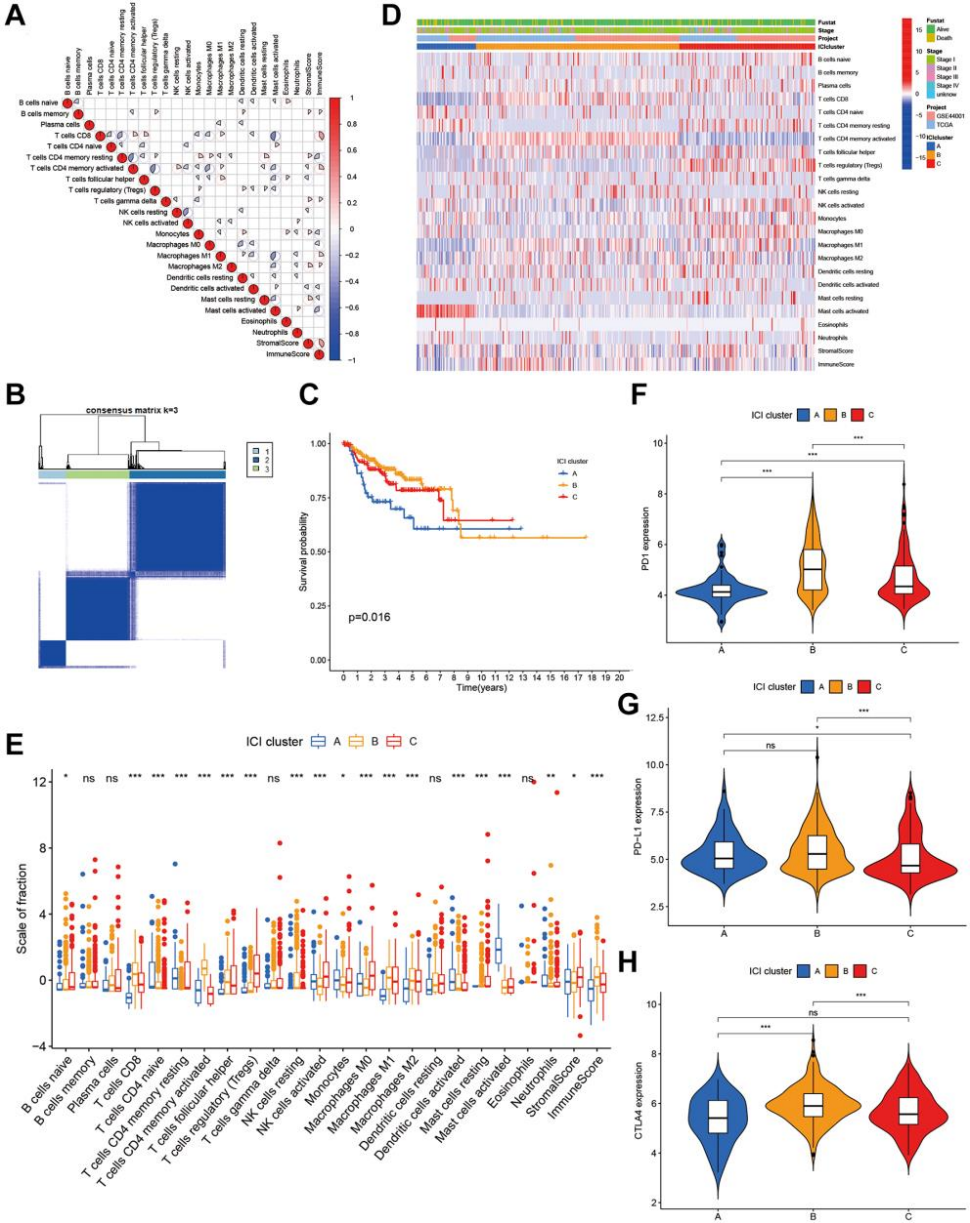
**The landscape of immune cells in the TME of CC.** (**A**) Correlation coefficient heatmap of the infiltrating immune cell types. (**B**) Unsupervised clustering of immune cells. (**C**) Kaplan-Meier curves of immune cell clusters. (**D**) Immune cell infiltration heat map. (**E**) The composition of immune cells in three ICI clusters. (**F**–**H**) The difference in PD1 (**F**), PD-L1 (**G**) and CLTA4 (**H**) expression among three ICI clusters.

We calculated infiltration contents of the 22 immune cells of each sample. Figure 1A visualized the correlation interactions of different immune cells in TME. The ConsensusClusterPlus R package was used for sample clustering, and 3 ICI subtype classifications were obtained (Figure 1B).

We carried out prognosis analysis on these three ICI subtypes and found that patients with ICI subtype A had a poor prognosis (*p* = 0.016, Figure 1C). To better clarify intrinsic differences of ICI subtypes, infiltration characteristics of immune cells were investigated in ICI subtypes (Figure 1D). ICI cluster A, which is related to a poorer prognosis, is characterized by low infiltrations of CD8+ T cells, macrophages M1, macrophages M2 and immune scores. It is also featured by high infiltration of activated mast cells (Figure 1E). The expression profiles of three major immune checkpoints (PD1, PD-L1 and CTLA-4) were displayed in Figure 1F–1H among three ICI subtypes (Figure 1F–1H).

### Immune gene subtypes

**Figure 2:**
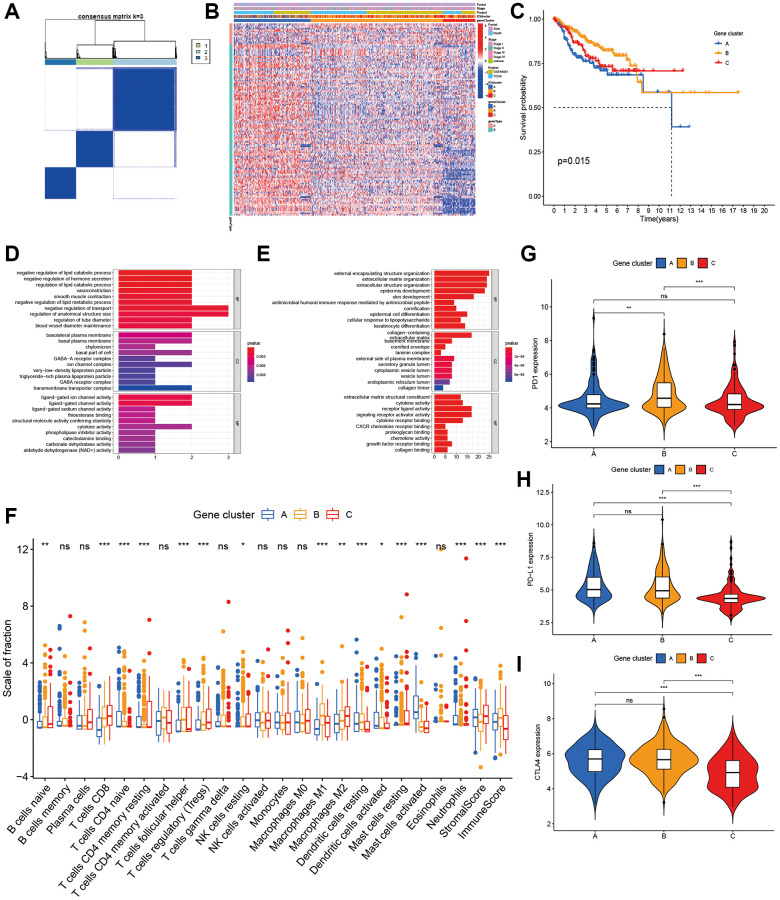
**Characterization of ICI Gene clusters.** (**A**) Cluster results of DEGs. (**B**) Heat map of DEGs characteristics. (**C**) Kaplan-Meier curves of CC patients with DEGs classes. (**D**, **E**) GO analyses of ICI signature genes A (**D**) and B (**E**). (**F**) The immune cell component in three gene clusters. (**G**–**I**) The difference in PD1 (**G**), PD-L1 (**H**) and CLTA4 (**I**) expression among three ICI gene clusters.

To prepare for the construction of ICI score model, unsupervised cluster of 129 DEGs was performed using the ConsensusClusterPlus package to obtain gene clusters (Supplementary Table 1). After the unsupervised cluster, the samples were re-classified into three ICI gene clusters (Figure 2A). Next, DEGs were distinguished based on positive or negative correlation with gene clusters. Of the 129 DEGs, the 115 that had inverse correlation with the gene cluster were assigned into the signature gene type B. The remaining DEGs were assigned into gene type A. The heatmap revealed the expression of DEGs in clinical features among samples (Figure 2B). Gene functional analysis was done using gene ontology (GO) enrichment on these gene types A and B (Figure 2D, 2E).

Next, we performed the Kaplan–Meier analysis on these 3 gene clusters, subjects in gene cluster A had a poor prognosis (*p* = 0.015, Figure 2C). As a guide from the results, gene cluster A was characterized on the low infiltration of CD8+ T cells, activated memory CD4 + T cells, and M1 macrophages, as well as low immune score and stromal score. It was also characterized on high infiltration of activated mast cells (Figure 2F). The expression of the 3 immune checkpoints in 3 gene clusters was shown in Figure 2G–2I.

### Calculation of ICI score

**Figure 3:**
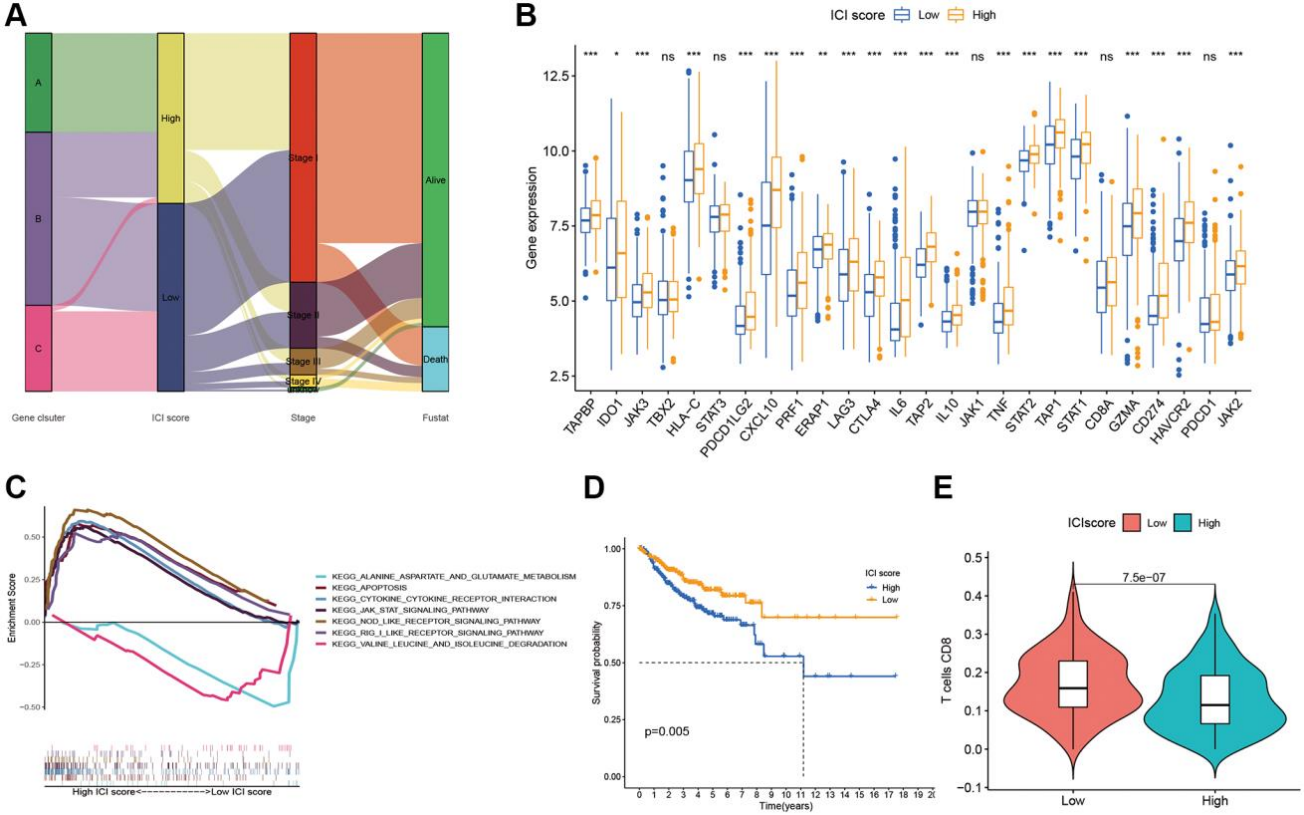
**ICI Scores construction.** (**A**) Sankey plot of ICI gene cluster, ICI scores, Stages, and OS. (**B**) Difference of immune-checkpoint, immune-activity-associated genes signature, and antigen presentation-related gene signature expression in the two ICI score subgroups. (**C**) Function pathways of ICI scores groups. (**D**) Kaplan-Meier plots of two ICI score groups. (**E**) The fraction of CD8+T cells in the two ICI score subgroups.

After quantifying individual ICI scores with PCA, the cases were subsequently assigned into low or high score groups. A Sankey diagram was used to visualize patients’ distributions of patients with gene clusters, stage, ICI scores, and survival status (Figure 3A). To determine immune activity and tolerance, we selected related signature genes with immune checkpoint, immune activity, and antigen presentation (Figure 3B). Biological differences between the ICI score groups were determined through gene set enrichment analysis (GSEA). These pathways included RIG-I-Like receptor signaling, apoptosis, NOD-Like receptor signaling, JAK-STAT signaling, cytokine-receptor interactions (Figure 3C). Patients with different ICI scores showed significant survival differences, with low ICI scores indicating better survival (Figure 3D).

### Tumor mutation burden (TMB)

**Figure 4:**
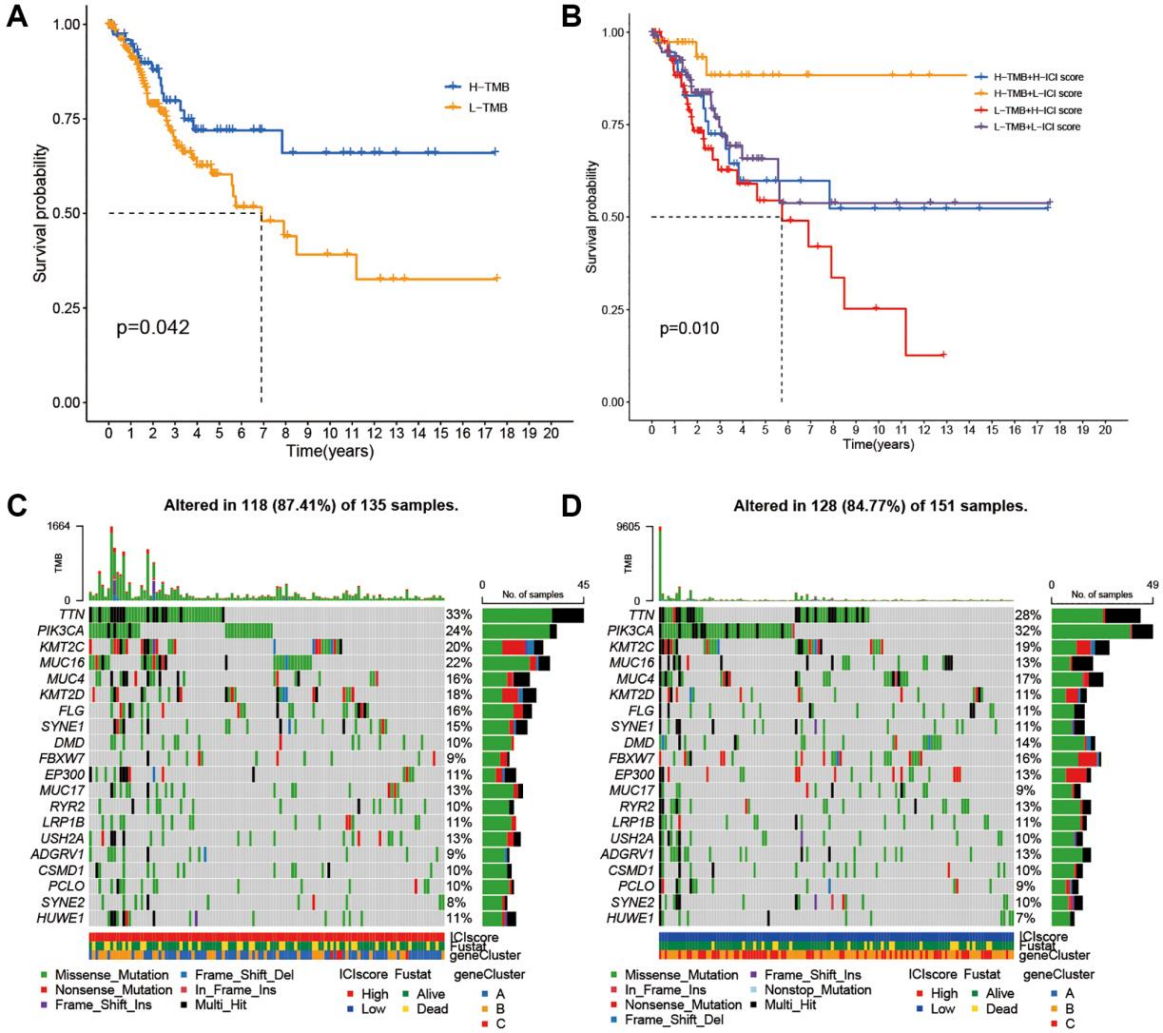
**Correlations between TMB and ICI Score.** (**A**) Survival plots of two TMB subgroups. (**B**) Survival plots combined with TMB and ICI scores. (**C**) Distribution of gene mutation in the high ICI score group. (**D**) Distribution of gene mutation in the low ICI score group.

Considering correlations of high TMB with prolonged progression-free survival, we attempted to detect an intra-association between TMB and ICI scores. Firstly, patients were assigned into low and high TMB groups based on optimal TMB cutoff values. As the Figure 4A showed that individuals with the high TMB had better overall survivals (OS). To discover whether the synergistic effect of TMB and ICI score exists in prognostic value, we conducted stratified survival analysis and found that the prediction value of ICI score was not interfered by the TMB. Two ICI score subtypes showed significantly different OS in both high and low TMB subgroups (Figure 4B). In summary, these findings suggest that ICI scores can be considered as a latent predictor independent of TMB.

Next, we compared the distribution of CC driver genes derived by Maftools between the two ICI score groups [19]. The top 20 driver genes with the most frequent alterations were further analyzed (Figure 4C, 4D). Table 1 showed that alteration frequencies of KRAS, MED12, TP53, PKHD1L1, and SPTA1 were significantly different within two ICI score subgroups.

**Table 1:** Distribution characteristic of somatic variations in the ICI scores.

**Gene**	**H-wild**	**H-mutation**	**L-wild**	**L-mutation**	***p*-value**
KRAS	134 (99.26%)	1 (0.74%)	137 (90.73%)	14 (9.27%)	0.003026
TP53	131 (97.04%)	4 (2.96%)	134 (88.74%)	17 (11.26%)	0.013975
PKHD1L1	121 (89.63%)	14 (10.37%)	147 (97.35%)	4 (2.65%)	0.01467
MED12	132 (97.78%)	3 (2.22%)	138 (91.39%)	13 (8.61%)	0.036738
SPTA1	132 (97.78%)	3 (2.22%)	138 (91.39%)	13 (8.61%)	0.036738

### Associations between ICI score and clinical features

**Figure 5:**
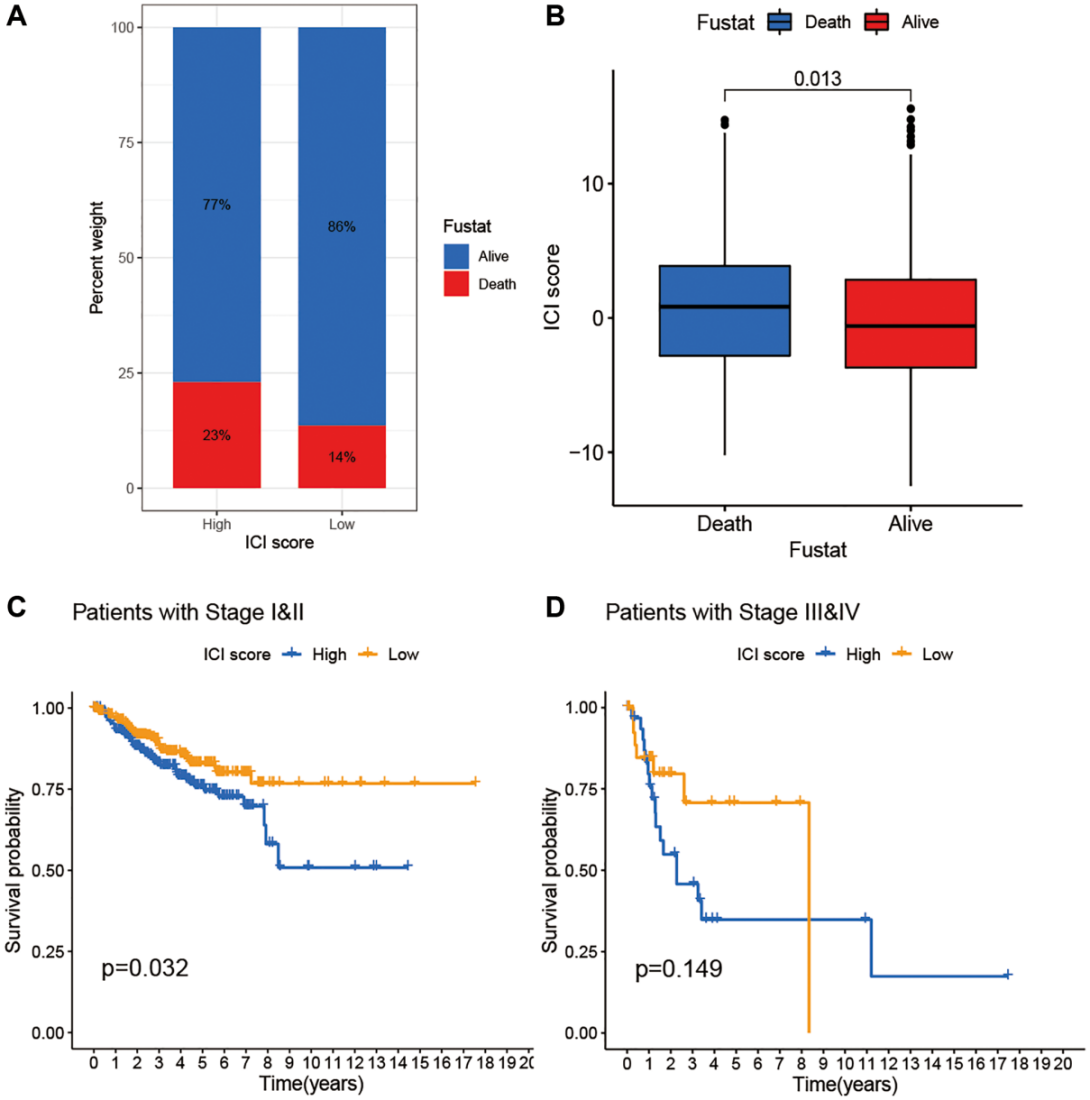
**The association of ICI scores and clinical characteristics.** (**A**) Rate of survival status of BC patients in the high and low ICI score group. (**B**) Distribution of survival status in the two groups. (**C**) Kaplan-Meier curves analysis for patients with Stage I and II. (**D**) Kaplan-Meier curves analysis for patients with Stage III and IV.

To assess potential clinical applications of ICI scores, we visualized distribution differences of survival status within two ICI score groups and found that patients who died had relative high ICI scores (Figure 5A, 5B). In addition, we performed survival analysis to evaluate prognostic implication of ICI score at different stages. Kaplan–Meier analysis showed that stages I and II patients with high ICI scores were correlated with worse prognosis than those with low scores. Furthermore, no significant difference was observed both in patients with high and low ICI scores at III and IV stages (Figure 5C, 5D).

### Immune escape

**Figure 6:**
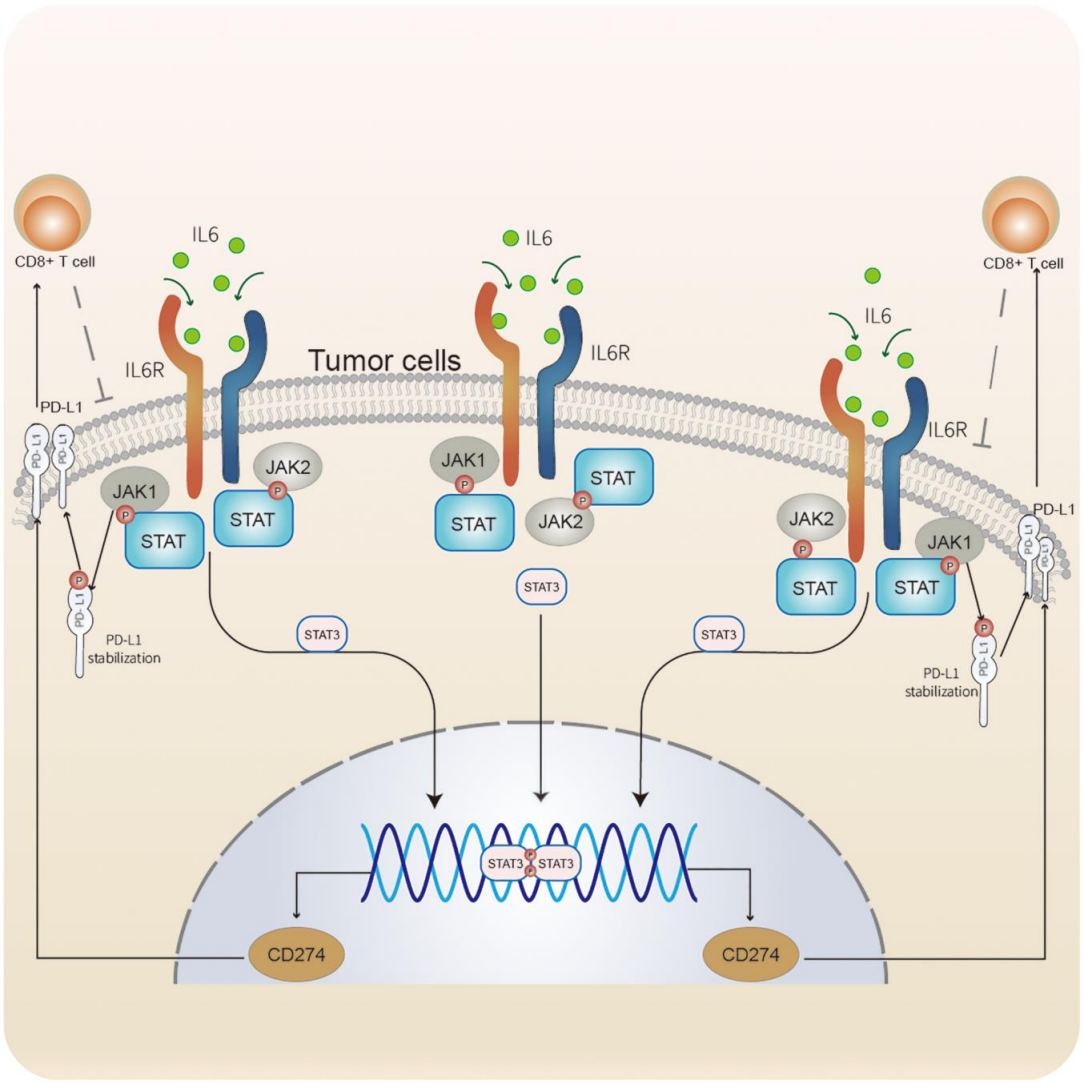
**Immune escape mediated by the IL6-JAK-STAT signaling pathway In CC.** IL6 binding to its receptors to activate the JAK-STAT pathway. JAK1 phosphorylates PD-L1 to stabilize PD-L1 protein. STAT3 was involved in regulating the transcription of CD274. PD-L1 modulates the expression and activation of CD8+ T cells. CD8+ T cells acting on tumor cells.

**Figure 7:**
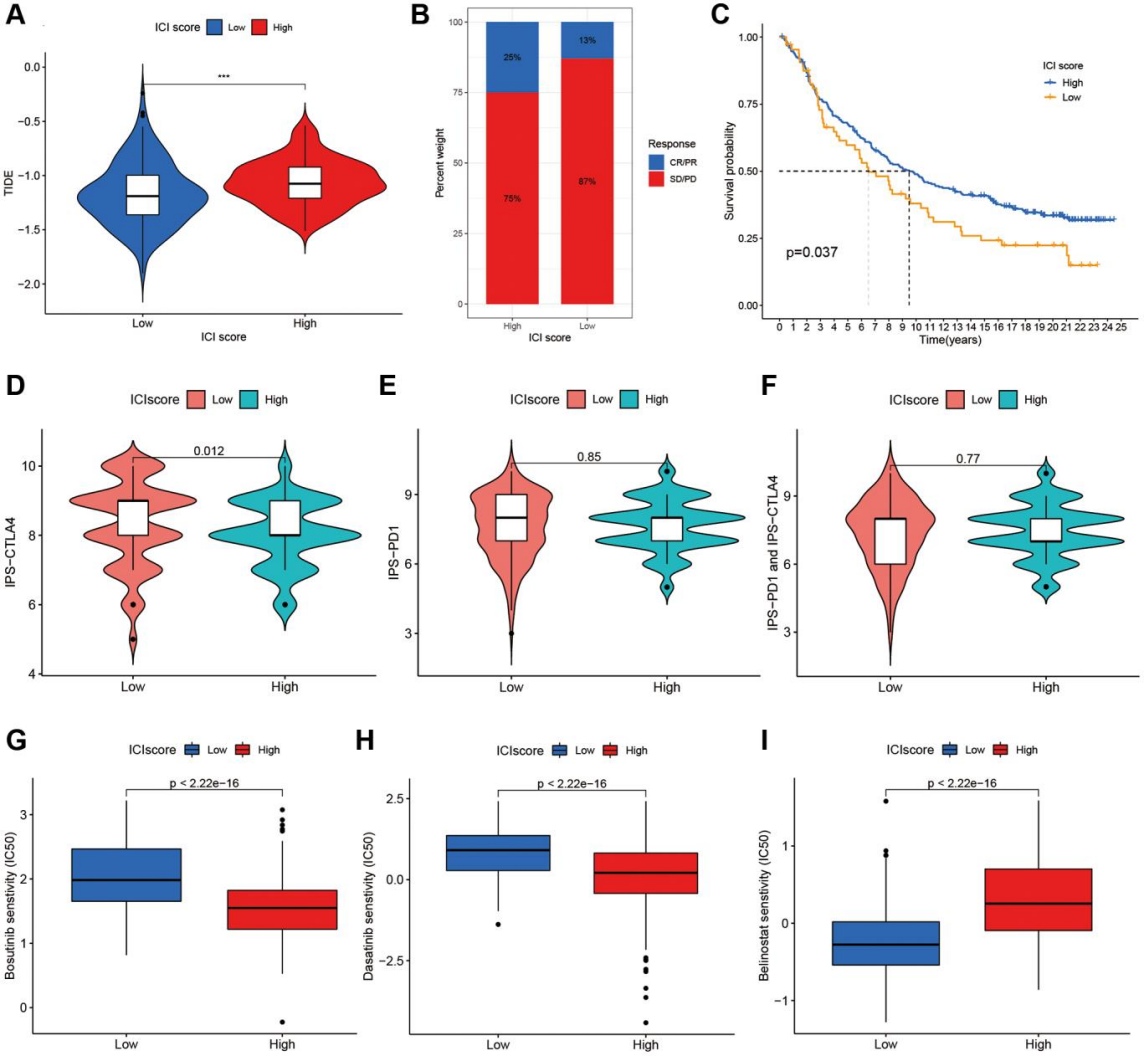
**The additional validation and prediction in immune escape and immunotherapy.** (**A**) The Additional Validation with TIDE scores in two ICI score group. (**B**) The immunotherapy response of anti-PD-L1 in the IMvigor210. (**C**) Survival plots of ICI scores in the IMvigor210. The responses of IPS-CTLA4 (**D**), IPS-PD1 (**E**) and IPS-PD1 and CTLA4 (**F**) in two ICI score groups. The sensitivities of Bosutinib (**G**), Dasatinib (**H**) and Belinostat (**I**) in two ICI score groups.

Through GSEA, we identified an underlying immune escape pathway, the JAK-STAT signaling pathways (Figure 3C), in high ICI score patients. Firstly, immune escape mediated by the JAK-STAT pathway in cancer had been demonstrated in prior studies [20, 21]. In our study, the escape mechanism depicting IL6-JAK-STAT3 signaling pathway had been illustrated in the Figure 6. The expressions of key factors and cells involved in this pathway, such as IL6, PD-L1, and CD8+ T cells, were compared between the two ICI score subgroups (Figure 3B, 3E) to discern whether the pathway functions in immune escape. Next, TIDE was used to verify the probability of immune escape (Figure 7A), and a higher TIDE score, a higher chance of immune escape. Our results showed that patients with high ICI score had a higher TIDE score and were more prone to immune escape.

### Immunotherapy

Immunotherapies by blocking T cell inhibition pathways (immune checkpoint blockade) had been widely applied to cancer treatment. To clarify responses to immunotherapy for different ICI score patients, we divided the IMvigor210 immunotherapy cohort patients, receiving the anti-PD-L1 intervention, into high or low ICI score groups using the ICI model. Patients with high ICI scores were observed higher response rates to anti-PD-L1 immunotherapy (Figure 7B) and better prognostic survivals (Figure 7C). Also, an additional TCIA database was used to further validate immunotherapy responses to PD1 and CTLA4 among individuals with different ICI scores. Figures 7D–7F displayed treatment responses to IPS, IPS-PD1, and IPS-CTLA4 in both ICI score group patients. Based on the pRRophetic method, we predicted the therapeutic sensitivity of TIs (Bosutinib, Dasatinib and Belinostat) between the two groups, and found two TIs (Bosutinib and Dasatinib) had higher IC50 in low score patients (Figure 7G, 7H). Individuals with low ICI scores were more sensitivity to Belinostat than those with high scores (Figure 7I).

## DISCUSSION

In our study, we explored the underlying mechanism of immune escape and investigated whether benefits to immunotherapy would be suitable for all patients with cervical cancer. In the process of tumor immune escape, tumor cells would overexpress PD-L1, which binds to PD-1 on the T cell surface to induce T cell exhaustion, failing to kill cancer cells and leading to immune escape. To the best of our knowledge, not all patients with cervical cancer are potential candidates for the universal immunotherapy protocols. To clarify the difference in expression of high and low ICI score patients and to solve the issue of immune escape, we performed comparisons of two key factor expressions, PD - L1 and CD8+ T cell, within the two ICI score patients and investigated the potential escape pathway through the GESA analysis. For different ICI score patients, individualized treatment regimens were analyzed and validated by the IMvigor210 and TCIA databases. Our results showed that high ICI scores patients could benefit more from anti-PD-L1 immunotherapy, and individuals with low scores may be better candidates for the anti-CTLA-4 treatment. Combinations of immunotherapies with targeted inhibitors may improve clinical efficacy and reduce the risk of tumor recurrence.

Due to the individual heterogeneity in the immune microenvironment, we developed the quantification model of the ICI score for comprehensive evaluation on outcomes. Using GSEA, we identified pathways related to immune responses, such as JAK-STAT signaling, NOD-like receptor signal, RIG-I-like receptor signaling, cytokine-cytokine receptor interaction, and apoptosis pathways. In particular, the IL-6- JAK-STAT3 signaling pathways plays a crucial role in human cancer [20]. Recently, a review by Kobayashi Y et al. detailed the immune escape mechanism of the JAK-STAT signaling pathway in cancers [21]. This pathway could be frequently activated in cancers or triggered by cytokines such as ILs [20–22]. In some cases, the JAK-STAT pathway may also be involved in resistance to chemotherapy or other treatments. Upon binding of cytokines to their cognate receptors, STATs could be activated by members of the JAK family of tyrosine kinases. Once activated, they dimerize and translocate to the nucleus and regulate the expression of target genes. Corresponding to the above, IL6 was significant higher in the high ICI score group, and the JAK-STAT signaling pathway was enriched in the same group. A similar expression was observed on PD-L1 within the two groups. Regarding PD-L1, it was regulated via a variety of signal pathways [23], one of these pathways is the JAK-STAT signaling [24]. PD-L1 could inhibit cytotoxicity and activation of CD8+ T cells and reduce the ratios of CD8+ T cells in the TME [25, 26]. In tumor immunity, CD8+ T cells act as the key tumor-suppressing cells and induce the death of tumor cells [27]. Deficiency of CD8+ T cells could not function their immune function and contribute to immune evasion of tumor cells [27, 28]. Furthermore, TIDE, a computational framework, was used to validate the probability of tumor immune escape. A higher TIDE score, a higher chance of immune escape and a worse immunotherapy response [29]. In the results section, we also reported patients in the high ICI score group had higher TIDE scores. Overall, patients with high ICI scores had a greater potential for immune escape due to higher TIDE scores and fewer CD8+ T cells.

For immunosuppressive checkpoints, expressions of PDCD1LG2, HAVCR2, and LAG3 were higher in the high ICI score group than those in the low, thereby suggesting that patients with high ICI scores could be more likely to benefit from immunotherapy. As shown in prior studies, genomic instability could affect immunotherapy by producing immune response phenotypes, and TMB as a predictor of immune checkpoint blockade could determine the possible response to immunotherapy drugs [30]. In the ICI score subgroups, we explored the frequencies of TMB in depth and found differences of gene mutation frequencies. Thus, patients with different ICI scores may be required to customize distinct immune-related drugs.

By complying with the individualized treatment regimens in immunotherapy, ICI scores were set to identify which patients would benefit from potential treatment regimens. As a promising approach, some drugs by blocking checkpoint, such as programmed cell death protein 1 (PD-1), PD-1 ligand (PD-L1), or cytotoxic T lymphocyte antigen-4 (CTLA-4), achieved notable efficacies in clinical treatments. Currently, Atezolizumab (antibody to PD-L1) and Ipilimumab (antibody to CTLA-4) were approved for cancer treatment. IPS acts as a superior predictive role in response to CTLA-4 and PD-1, and the higher the IPS, the better response to PD-1 and CTLA-4 [17]. According to the results from the IMvigor210 and TCIA, patients with the high ICI scores may be better candidates for the anti-PD-L1 therapy, and the anti-CTLA-4 protocol was more suitable for the remaining patients. In this study, a large quantity of TIs had exhibited distinct difference in sensitivity between ICI score subgroups. IC50 was used to infer the sensitivity of the different patients, and the lower the IC50, the more sensitive to drugs [18, 31]. Of all TIs, Bosutinib and Dasatinib were screened out as targeted inhibitors associated with the JAK-STAT signaling. Belinostat was selected as a potential candidate drug for tumor relapse. Interestingly, the JAK-STAT signaling had been demonstrated as a vital regulator of cancer stem cells (CSCs) and involved in the recurrence of tumors mediated by CSCs [32, 33]. Therefore, combination therapies may offer more efficient than single immunotherapy for relapsed patients. For patients with high ICI scores, combinations of the anti-PD-L1 and targeted inhibitors (e.g., Bosutinib) in the clinical treatment may be helpful for prevention of tumor recurrence. A combination of anti-CTLA-4 with targeted inhibitors (e.g., Belinostat) may improve the efficacy and reduce recurrence risk for patients with low ICI scores. Thus, this study speculated that patients with different ICI scores can benefit from individualized treatment regimens in immunotherapy. However, the therapeutic efficacy on patients with cervical cancer still deserves further investigation in depth. Additionally, stratification analysis suggested that the index of ICI score was independent of TMB in prognosis of cervical cancer, which contributing to guiding individualized treatment regimens in immunotherapy.

The limitations are as follows: An additional experiment *in vivo* to investigate the immune escape is required to further validation and more independent cohorts of immunotherapy are warranted for validation of the reliability and stability of the ICI score model.

In conclusion, we comprehensively analyzed the ICI landscape in CC and established an ICI score model to evaluate the immune escape and immunotherapy. Benefit from the ICI score model, we revealed the underlying immune escape mechanism that providing a clear immune escape pathway and predicted the response to immunotherapy in different populations. Therefore, ICI score model played an important role in clinical significance and customized the optimal immunotherapy strategy for the target candidates.

## Supplementary Materials

Supplementary Table 1
